# AlterNet-K: a small and compact model for the detection of glaucoma

**DOI:** 10.1007/s13534-023-00307-6

**Published:** 2023-07-30

**Authors:** Gavin D’Souza, P. C. Siddalingaswamy, Mayur Anand Pandya

**Affiliations:** 1 Department of Instrumentation and Control Engineering, Manipal Institute of Technology, Manipal Academy of Higher Education, Manipal, Karnataka 576104 India; 2 Department of Computer Science and Engineering, Manipal Institute of Technology, Manipal Academy of Higher Education, Manipal, Karnataka 576104 India

**Keywords:** Glaucoma, ResNet, Deep learning, CNN, Medical imaging, Classification, Transformer

## Abstract

Glaucoma is one of the leading causes of permanent blindness in the world. It is caused due to an increase in the intraocular pressure within the eye that harms the optic nerve. People suffering from Glaucoma often do not notice any changes in their vision in the early stages. However, as it progresses, Glaucoma usually leads to vision loss that is irreversible in many cases. Thus, early diagnosis of this eye disease is of critical importance. The fundus image is one of the most used diagnostic tools for glaucoma detection. However, drawing accurate insights from these images requires them to be manually analyzed by medical experts, which is a time-consuming process. In this work, we propose a parameter-efficient AlterNet-K model based on an alternating design pattern, which combines ResNets and multi-head self-attention (MSA) to leverage their complementary properties to improve the generalizability of the overall model. The model was trained on the Rotterdam EyePACS AIROGS dataset, comprising 113,893 colour fundus images from 60,357 subjects. The AlterNet-K model outperformed transformer models such as ViT, DeiT-S, and Swin transformer, standard DCNN models including ResNet, EfficientNet, MobileNet and VGG with an accuracy of 0.916, AUROC of 0.968 and F1 score of 0.915. The results indicate that smaller CNN models combined with self-attention mechanisms can achieve high classification accuracies. Small and compact Resnet models combined with MSA outperform their larger counterparts. The models in this work can be extended to handle classification tasks in other medical imaging domains.

## Introduction

Glaucoma is a leading cause of permanent visual impairment worldwide. In 2022, Glaucoma affected around 80 million individuals, and experts project that this number will rise to 112 million by 2040 [1, 2]. Glaucoma is mainly caused by a high intraocular pressure (IOP) inside the eye that harms the optic nerve. However, it can also occur at normal levels of IOP and more frequently in patients who have diabetes [2, 3]. An early symptom of Glaucoma is the loss of peripheral vision. Visual signals are transmitted to the brain through the retinal nerves on the Optic Disc (OD), a circular area within the eye where the optic nerve connects to the retina. The OD also consists of photoreceptors that support vision. Besides photoreceptors, the OD contains a white area called the Optic Cup (OC) at its centre. A popular method for detecting Glaucoma involves calculating the vertical (along diameter) cup-to-disc ratio. An abnormally sizeable cup-to-disc ratio indicates that the patient suffers from Glaucoma [3, 4]. Standard non-invasive medical imaging methods used to detect Glaucoma are fundus imaging and Optical Coherence Tomography (OCT). Fundus cameras can change filters to improve the fundus images taken at various angles, allowing for precise and detailed retina views to be acquired. However, drawing accurate insights from these images requires them to be manually analyzed by medical experts, which is a time-consuming process. As such, the non-automated glaucoma diagnosis process is lengthy, expensive, and unscalable. It is, therefore, necessary to have Computer-aided Diagnostic systems (CAD) capable of performing initial screenings to reduce the burden on professionals.

Automatic Glaucoma detection techniques can be separated into heuristic and deep learning methods [5]. Heuristic methods rely on custom handcrafted filters and image processing techniques for feature extraction. These features are passed to support vector machines (SVM) or Bayesian Classifiers. These methods are not entirely accurate as they consider just a small number of features (handcrafted) on fundus images. Developing these handcrafted feature extractors relies on the prior knowledge of an expert in the field.

In contrast, deep learning methods offer more robust, scalable, and highly accurate solutions. Traditional heuristic techniques have been significantly outperformed by Deep Learning, a branch of machine learning that employs brain-inspired algorithms called Artificial Neural Networks (ANN). Particularly the Convolutional Neural Network (CNN) and its ability to learn to extract high-level feature representations from image data have shown excellent results. These models have exhibited reliable results across diverse computer-vision fields, encompassing classification, segmentation, and image generation. They have been rapidly adopted for medical image processing and have set new benchmarks due to their scalable nature and the availability of large datasets.

In recent years, the multi-head self-attention (MSA) based Transformer architecture [6], developed for sequence processing in NLP, has gained widespread traction in vision and speech domains due to its modality-agnostic nature. Due to their ability to capture long-range dependencies, attention mechanisms help to improve performance in various computer vision tasks. In particular, the Vision Transformer (ViT) [7], a pure transformer architecture applied directly to sequences of non-overlapping image patches, showed competitive performances with CNN models such as ResNet [8]. However, these transformer models must be pre-trained on enormous amounts of data to achieve competitive results. Several recent works [9, 10] explore the effectiveness of modern transformer architectures based on ViT in diagnosing retinal diseases.

Deep learning methods for medical image classification mostly employ ensemble learning strategies with large DCNN models to boost performance. Developing large models and making inferences involves significant computation costs. However, it is worth noting that even with these costs, larger models trained on small datasets may not necessarily perform better than smaller ones. This work proposes an efficient and compact model for Glaucoma detection. The model is evaluated on the Rotterdam EyePACS AIROGS dataset [11] and other popular models used for Glaucoma detection.

## Literature review

The application of deep learning to medical imaging for eye diseases has been the subject of extensive research. Fundus images are one of the most used techniques to get a clear and precise retina view. Several works explore the effectiveness of deep learning in the classification of colour fundus images into referable/non-referable glaucoma samples [3, 12–14]. Diaz-Pinto et al. [3] presented five different CNN architectures for glaucoma assessment namely, ResNet50, VGG16, VGG19, InceptionV3 [15], Xception [16]. The models were pre-trained on the ImageNet dataset and further fine-tuned on publicly available glaucoma datasets. They carried out a series of experiments to determine the optimal number of epochs and fine-tuned layers. Performance was assessed as a trade-off between the AUC score and parameter count, with the Xception architecture demonstrating the best results. Akkara et al. [12] suggested a 13-layer CNN architecture containing 10 CNN layers and 3 fully connected layers. SoftMax and Support Vector Machine (SVM) classifiers were used to classify images. The accuracy of the CNN with SoftMax classifier was 93.86%, whereas CNN with an SVM classifier attained an accuracy of 95.61%. Images gathered from multiple public datasets and a private research centre comprised the dataset used for this investigation. The total number of photos in the test set was 114. Li et al. [13] proposed a method for glaucoma classification that considers both local and global features in the image. The method involves learning features from a selected region of interest (ROI) centred on the optic disc in the image. During the feature extraction process, the ROI image is split into a 3 × 3 grid of patches. Features are extracted independently using a CNN from these patches and the whole ROI. Two independent classifiers are trained using both sets of features, and the decision values are combined using a weighted sum. Similarly, Al-Mahrooqi et al. [17] proposed a multi-view system composed of 3 independent networks that each process a different view of the image. These views include the full-resized image; the ROI cropped around the OD, and a polar transformation of the cropped ROI. They trained a U-Net model for the segmentation of the ROI region. The method achieved an AUC score of 0.92 on the Rotterdam EyePACS AIROGS dataset. Khader et al. [18] described their approach for glaucoma detection in the Artificial Intelligence for Robust Glaucoma Screening challenge. They trained a YOLO object detector to extract the ROI around the OD, followed by an ensemble of four networks to process the extracted ROI independently. The ensemble consisted of EfficientNetV2-M, EfficientNet-B4, Swin transformer-B and DeiT-S. Their method achieved a partial AUC score of 0.8884 on the Rotterdam EyePACS AIROGS dataset.

Wang et al. [14] suggested an ensemble strategy where two models are trained on RGB and greyscale fundus images separately. Each model consisted of an EfficientNet feature extractor and a custom classification network. The final output was computed by averaging the output probabilities of both models. Hemelings et al. [4] applied an inverse cropping mask to the optic disk within all images and trained a ResNet50 model on these images cropped images, which no longer contained the Optic Nerve Head (ONH). They trained a second model on the original images and compared their AUC scores. They found that both models achieved similar AUC scores, 0.88 and 0.94, corresponding to the first and second models. This showed that the model could learn features outside the ONH for glaucoma detection. Another popular deep-learning approach for Glaucoma identification involves a direct investigation of the quantitative aspects of the OD and OC. Shanmugam et al. [19] trained a segmentation network to segment the areas of the OD and OC. The cup-to-disc ratio (CDR) from these segmented regions is computed and fed to a random forest classifier. Various segmentation models (Au-Net, deformable U-Net, Full-Deformable U-Net, and original U-Net) were evaluated for the segmentation step, with Au-Net giving the best results. They verified their methods on the DRISHTI-GS dataset.

Li et al. [5] presented the AG-CNN, an attention-based technique for detecting and localizing Glaucoma. The model comprises three sub-networks for attention prediction, pathological area localization and glaucoma classification. Self-attention is the central feature of transformer architecture [6]. Fan et al. [9] performed a study to compare the generalizability of the SoTA Transformer model Data-efficient image Transformer (DeiT) [20] with the classical ResNet-50 CNN model for detecting glaucoma in Ocular Hypertension Treatment Study (OHTS) photographs [21]. They found that the DeiT model performed similarly to ResNet50 on OHTS. Furthermore, testing on external independent datasets containing fundus photographs from people of various ethnicities, including African, Chinese, Japanese, European, and Spanish, showed that DeiT outperforms ResNet-50.

Park and Kim [22] performed an extensive study of the properties of MSA. They showed that, in multi-stage CNN networks, MSA at the end of a stage significantly improves performance. Moreover, Convs acted as high-pass filters, while MSAs acted as low-pass filters. They showed that MSAs tend to improve generalization by flattening loss landscapes. Moreover, when combined appropriately, Convs and MSAs alleviate each other’s limitations due to their complementary properties. Park and Kim [22] proposed a novel architecture called the AlterNet built by alternatively substituting MSA blocks for Conv blocks beginning at the end of a baseline model. The AlterNet based on pre-activation ResNet50 outperformed standard resnet50 on the CIFAR-100 dataset. Li et al. [23] proposed a novel vision transformer called Swin-Transformer that serves as a transformer backbone for computer vision tasks. Li et al. [23] proposed the Swin Transformer, a novel vision transformer that functions as a transformer backbone in vision models. Unlike traditional ViTs, this model computes attention using shifting windows rather than global attention. This shifted window method results in better efficiency by focusing self-attention on small, local groups of patches. He et al. [10] proposed a network that combined Swin-Transformer and PolyLoss [24] to diagnose several retinal diseases in OCT images automatically. To visually understand the decision-making ability of the model, they create Class Activation Mappings (CAMs) using the score-CAM [25] method. Overall, the method outperforms ViT and the regular Swin-Transformer model by achieving an AUROC score of 0.9999 on the OCT2017 [26] dataset and an AUROC score of 0.9962 on the OCT-C8 dataset [27].

Earlier works on retinal image processing mostly employ large CNN networks for feature extraction, followed by ensemble learning techniques to boost performance. These ensemble methods significantly increase computation costs during training and inference. Most methods usually rely on multiple views of the image to improve prediction performance [3, 17–19]. They employ separate localization networks that extract the ROI containing the ONH as a pre-processing step. This often requires manual labelling of the region surrounding the optic disc. Further, to obtain high accuracy, several independent networks are trained to process these views separately. Works like Hemelings et al. [4] showed that deep learning models could learn to detect Glaucoma even if the OD is cropped out of the fundus image. Very few works study the effectiveness of attention mechanisms incorporated with CNNs for medical image tasks. Therefore, this work aims to use the complementary natures of CNNs and MSAs to develop a parameter-efficient model for Glaucoma identification that accurately detects Glaucoma without requiring external ROI extraction algorithms. We compare the performance of our model to modern transformer architectures, including ViT, Swin Transformer and DeiT. Further, we compare it with standard DCNN models such as ResNet, EfficientNet [28], VGG [29] and MobileNet [30, 31]. In summary, the main contributions of this work are as follows:

1. We develop a parameter-efficient model called AlterNet-K for Glaucoma identification that accurately detects Glaucoma without requiring external ROI extraction algorithms.
2. We show that limiting the maximum channel length throughout the AlterNet-K model can improve the model’s performance in Glaucoma detection while reducing the number of parameters.
3. We evaluate our model and several SoTA Transformer and classical CNN models on the Rotterdam EyePACS AIROGS dataset.

## Materials and methods

**Fig. 1:**
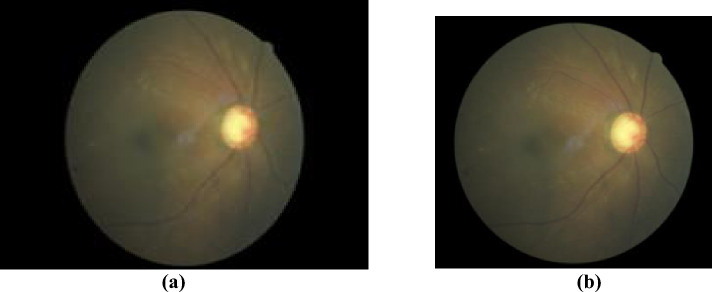
**a** Resized image with aspect ratio > 1.3, **b** resized image with aspect ratio = 1.1

This study uses the Rotterdam EyePACS AIROGS dataset, comprising 113,893 colour fundus images from 60,357 subjects, as a part of the Artificial Intelligence for Robust Glaucoma Screening (AIROGS) challenge [11]. The dataset is split into two sets: a training set containing 101,442 images and a test set with about 11,000 images. The test set, however, is not publicly available. As such, we had to use our own train and validation splits. This limited our ability to compare our results with other works that used unique dataset partitions. The dataset is imbalanced with 98,172 images manually labelled as “No Recordable Glaucoma” (NRG) class as compared to the mere 3270 images labelled as “Recordable Glaucoma” (RG) class. 3270 NRG images are randomly chosen from the original 98,172 to create a balanced subset. Therefore, the selected subset comprises 6540 images equally divided among the positive (RG) and negative classes (NRG). NRG and RG labels are encoded to 0 and 1, respectively. The images are resized using the Python PIL package to 224 × 224. The images in the dataset have aspect ratios ranging from (1 to 1.8). Images with high aspect ratios appear to be squeezed when resized directly to new dimensions with an aspect ratio of 1. Therefore, the images are cropped to reduce the aspect ratio to 1.1 before resizing. Figure 1a shows an image with a high aspect ratio after resizing, and Fig. 1b shows an image with a reduced aspect ratio. Each model is evaluated using fivefold cross-validation. The Python programming language and the PyTorch library were utilized for data processing and model training in Google Colaboratory. Pretrained Transformer models were obtained from the HuggingFace Transformers library. The Python code utilized in this study is accessible in the https://github.com/gavin-d26/AlterNet-K-A-Small-and-Compact-Model-for-the-Detection-of-Glaucoma upon request.

## Methodology

### AlterNet-K architecture

This section presents the AlterNet-K model architecture, a variant of the model proposed in Park and Kim [22]. Park and Kim [22] showed that Convs and MSAs have complementary properties. MSAs tend to dampen the high-frequency components in the image, whereas Convs in ResNet tend to amplify them. Low-frequency signals correspond to the shapes of objects within images, while higher-frequency signals correspond to the textures in the image. Therefore, MSAs are shape-biased, while Convs are texture-biased. A suggested improvement for baseline CNN models is to replace Conv blocks with MSA blocks, alternating them from the end.

**Fig. 2:**
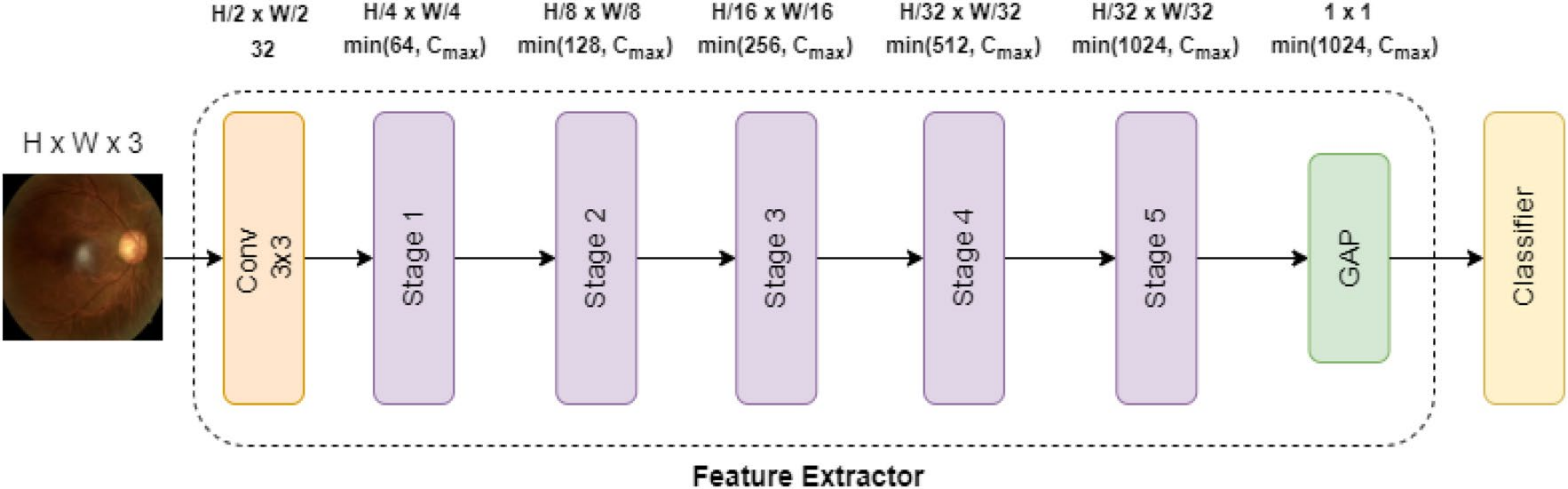
Architecture of AlterNet-K

The AlterNet-K is based on the alternating design pattern proposed in Park and Kim [22] and aims to (1) harmonizes MSAs with ResNets, and (2) minimize the number of parameters in the model. As shown in Fig. 2, AlterNet-K consists of an initial Conv layer with a channel length of 32 and a 2 × 2 max-pooling layer followed by five intermediate stages, each consisting of sequential connections of a ResNet block, MSA block and a 2 × 2 max pooling layer. The initial Conv layer and intermediate stages serve as the feature extraction module. A global pooling operation aggregates the feature extractor output and feeds it to a classifier consisting of a fully connected layer and a sigmoid function. To reduce the number of parameters in the model, we limit the maximum permissible channel length (${C}_{max}$) in all stages of the model. The “K” in AlterNet-K is an indicator of ${C}_{max}$ in all stages of the model. For example, AlterNet-K256 represents a model wherein ${C}_{max}$ in any stage is 256.

**Fig. 3:**
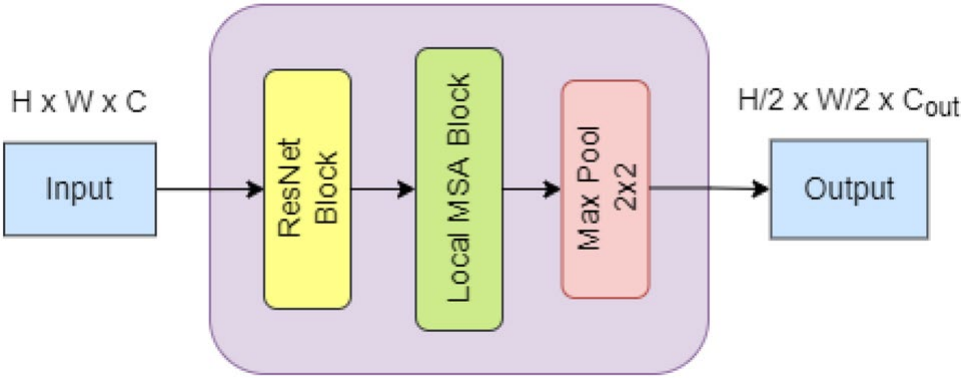
Architecture of an intermediate stage of AlterNet-K

As shown in Fig. 3, Each intermediate stage of the model follows an identical structure consisting of a sequential connection of a ResNet block, MSA block and a 2 × 2 max pooling layer. Unlike the AlterNet architecture proposed in Park and Kim [22] and standard ResNets such as ResNet50, AlterNet-K contains no residual bottleneck blocks. Each intermediate stage inputs feature maps with dimensions $H\times W\times C$ and produce output feature maps with dimensions $\frac{H}{2} \times \frac{W}{2} \times {C}_{out}$ where ${C}_{out}$= $min(2C$, ${C}_{max}$) and ${C}_{max}$ is the maximum permissible channel length in every stage of the model. This means that an intermediate stage that takes input feature maps of dimension $H \times W \times {C}_{max}$ produces output feature maps with dimensions $\frac{H}{2} \times \frac{W}{2} \times {C}_{max}$. This restriction on channel length improves the model’s performance on the limited-sized dataset while reducing the parameter count of the model. The last intermediate stage of the network does not include a max pooling operation. AlterNet-K with five intermediate stages and ${C}_{max}=128$, denoted as AlterNet-K128, gave the best classification performance.

**Fig. 4:**
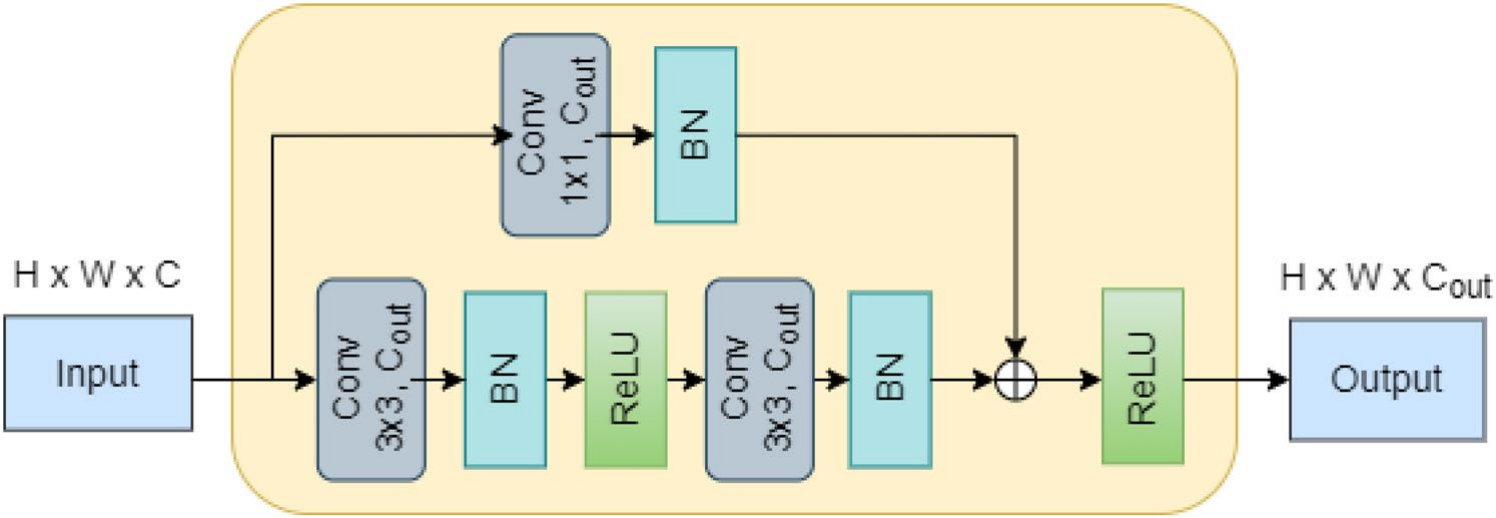
Architecture of a ResNet block in AlterNet-K

Figure 4 shows the architecture of the ResNet block used in each model stage. The ResNet block consists of two sequential Conv layers with batch normalization and ReLU activation. The channel length of the outputs of each of these blocks is ${C}_{out}$. The inputs are added to the output of the two sequential Conv nets via a residual skip connection. A non-activated $1\times 1$ Conv layer with the batch norm is used to resize the channel length of the input from $C$ to ${C}_{out}$, to match the dimensions of the output of the two sequential Conv layers. Finally, the residual output is activated by a ReLU activation function.

**Fig. 5:**
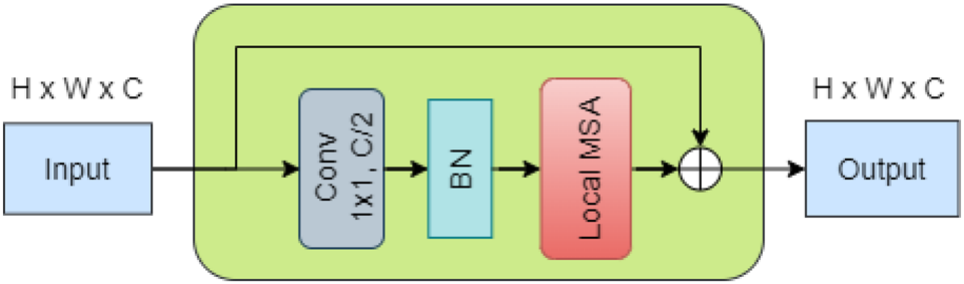
Architecture of MSA block in AlterNet-K

**Fig. 6:**
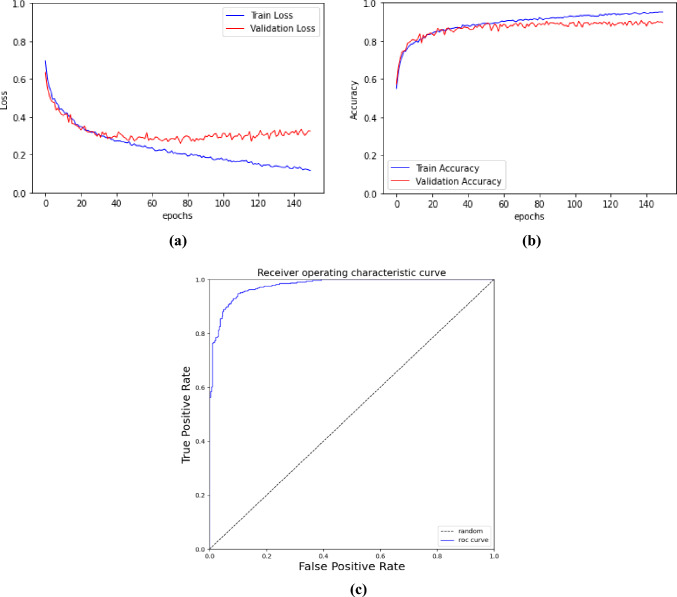
**a** AlterNet-K128 loss curves, **b** AlterNet-K128 accuracy curves, **c** ROC curve for AlterNet-K128 on the Rotterdam EyePACS AIROGS dataset

Figure 5 shows the internal structure of the MSA blocks that serve as trainable spatial smoothing layers. They enhance the model’s generalizability by reducing the variance of the feature maps produced by the ResNet blocks. A non-activated $1\times 1$ Conv layer followed by a batch norm layer is used to reduce the channel length of the features from $c$ to $\frac{C}{2}$ before the local MSA layer processes them. The original inputs to the MSA block are added to the output of the local MSA layer via a residual skip connection. As shown in Fig. 6, the output of the Local MSA layer has the exact dimensions as the original input, i.e., $H\times W\times C$. The dimension of each attention head in the local MSA layer is $d=16$. Therefore, the number of attention heads in the local MSA layer is $\frac{C}{2d}$. Transformers such as ViT and Swin [7, 23] divide the feature map into non-overlapping patches and use them to input their attention modules. AlterNet-K does not divide the feature map into patches and applies self-attention directly to every pixel in the feature map. In contrast to global MSA, which calculates self-attention across the entire feature map, our local MSA layer calculates attention within 7 × 7 non-overlapping local windows of pixels. The attention operation is performed separately for each window. These locality constraints on MSA improve computational efficiency and predictive performance compared to global attention [22, 23]. Since most of the discriminative features of Glaucoma are concentrated within a small portion of the image, i.e. within the OD, attending to a smaller window can prevent the model from being influenced by unrelated elements, leading to more robust predictions.

## Results and discussions

### Implementation

We compared the AlterNet-K model, specifically AlterNet-K128, with ${C}_{max}=128$, to various pre-trained transformer models such as ViT, DeiT-S (a smaller version of DeiT), Swin-T (Swin-tiny), and Swin-B (Swin-base) [9, 10]. The ViT and DeiT-S models used a patch size of 16 [7, 20], while Swin-T and Swin-B used a patch size of 4 and a window size of 7 [23]. Each model is trained and evaluated using PyTorch and its associated modules. The pre-trained ViT, Swin and Deit-S model weights were obtained from the HuggingFace Transformers library. They were originally pre-trained on the ImageNet-1 k dataset and then fine-tuned on the Rotterdam EyePACS AIROGS dataset in this work. All models were trained using Nvidia Tesla V100 GPUs in Google Colaboratory. We incorporated data augmentations during training to prevent overfitting, including random horizontal and vertical flipping (*p* = 0.5) and random rotation (− 30° to 30°). All models (except baseline transformer models) were trained from scratch with a standard set of hyperparameters using the Adam optimizer with beta values (0.9, 0.9) and a learning rate of 0.001. The binary cross-entropy loss function is employed to compute the loss. For the pre-trained Transformer models, we used a learning rate of 2e−4. The models are trained with a batch size of 32 and a dropout rate of 0.1 if applicable. We trained each model for 150 epochs, utilizing Cosine Annealing with warm restarts for models containing MSA and Reduce on Plateau learning rate scheduler for models without MSAs. We evaluated each model using fivefold cross-validation and reported the average performance of the model on all partitions. Further, we conduct an ablation study in Sect. 5.3 to determine the effect of MSAs in AlterNet-K.

### Classification results

Table 1 shows the performance metrics for the AlterNet-K128, Transformer and DCNN models. The AlterNet-K128 model outperforms all models in every metric. It achieved an accuracy of 0.9161 and a recall of 0.9070, outperforming both the Swin transformer and EfficientNet models. It is also the most memory efficient, comprising the least parameters. The AlterNet-K128 also outperforms the MobileNetV3_small model, the DCNN model with the least number of network parameters. It is also interesting to note that ResNet18 outperformed the larger ResNet34 and ResNet50 and was almost as accurate as the Swin Transformer models. We could not accurately compare with other works that used the same dataset [17, 18] as they used different train and test splits of the dataset. Figure 6a shows the epoch-wise training and validation loss of AlterNet-K128 during training. Similarly, Fig. 6b shows the training and validation accuracy during training. Figure 6c shows the ROC curve for the AlterNet-K128 model.

**Table 1:** A comparative analysis of AlterNet-K with commonly used Glaucoma Detection methods on the Rotterdam EyePACS AIROGS dataset

Model	Accuracy	AUROC	Precision	Recall	Specificity	F1 score	Parameter count
ViT	0.8841 ± 0.0057	0.9466 ± 0.0041	0.8911 ± 0.0092	0.8752 ± 0.0053	0.8930 ± 0.0102	0.8831 ± 0.0053	85,799,425
DeiT-S	0.8618 ± 0.0124	0.9286 ± 0.0130	0.8668 ± 0.0156	0.8554 ± 0.0238	0.8682 ± 0.0181	0.8608 ± 0.0133	21,666,049
Swin-T	0.8968 ± 0.0098	0.9524 ± 0.0052	0.9047 ± 0.0010	0.8872 ± 0.0165	0.9064 ± 0.0105	0.8957 ± 0.0103	27,520,123
Swin-B	0.8956 ± 0.0007	0.9525 ± 0.0013	0.9019 ± 0.0154	0.8881 ± 0.0206	0.9031 ± 0.0191	0.8947 ± 0.0030	86,744,249
ResNet18	0.8922 ± 0.0023	0.9559 ± 0.0045	0.9005 ± 0.0114	0.8823 ± 0.0157	0.9021 ± 0.0143	0.8911 ± 0.0032	11,177,025
ResNet34	0.8827 ± 0.0080	0.9512 ± 0.0050	0.8833 ± 0.0133	0.8826 ± 0.0266	0.8828 ± 0.0170	0.8825 ± 0.0096	21,285,185
ResNet50	0.8838 ± 0.0062	0.9518 ± 0.0053	0.9024 ± 0.0191	0.8615 ± 0.0186	0.9061 ± 0.0215	0.8811 ± 0.0060	23,510,081
EfficientNetB0	0.9023 ± 0.0057	0.9619 ± 0.0047	0.9183 ± 0.0113	0.8835 ± 0.0136	0.9211 ± 0.0122	0.9004 ± 0.0060	4,008,829
EfficientNetB1	0.9080 ± 0.0031	0.9642 ± 0.0031	0.9219 ± 0.0117	0.8917 ± 0.0088	0.9242 ± 0.0129	0.9064 ± 0.0025	6,514,465
MobileNetV3_small	0.8833 ± 0.0053	0.9479 ± 0.0052	0.8954 ± 0.0125	0.8685 ± 0.0138	0.8982 ± 0.0145	0.8816 ± 0.0055	1,518,881
VGG_11	0.8804 ± 0.0098	0.9468 ± 0.0077	0.8776 ± 0.0185	0.8853 ± 0.0302	0.8755 ± 0.0249	0.8809 ± 0.0111	116,186,881
AlterNet-K128 (Ours)	0.9161 ± 0.0058	0.9687 ± 0.0050	0.9239 ± 0.0113	0.9070 ± 0.0069	0.9251 ± 0.0122	0.9153 ± 0.0054	1,299,342

### Ablation study

**Fig. 7:**
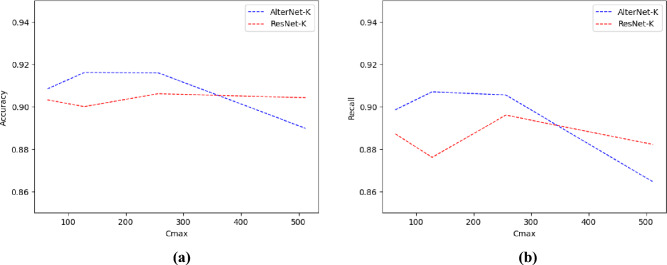
**a** Test accuracy v/s ${C}_{max}$, **b** Test recall v/s ${C}_{max}$

*Effect of MSA on AlterNet-K model performance* This section explores how MSA affects AlterNet-K performance. To evaluate this, we developed a new model, ResNet-K, with the same architecture as AlterNet-K but without the MSA layers in each stage. We compared the accuracy and recall metrics of AlterNet-K and ResNet-K at different values of ${C}_{max}$ as shown in Table 2. We plot these values in Fig. 7a, b. Figure 7a plots the accuracy v/s ${C}_{max}$ for each model, whereas Fig. 7b plots the recall v/s ${C}_{max}$ for each model. Our results indicate that AlterNet-K performs best when ${C}_{max}=128$, outperforming ResNet-K for all ${C}_{max}$ values. Additionally, our analysis in Fig. 7a, b demonstrates that the accuracy and recall of AlterNet-K are maximum for a ${C}_{max}$ value of 128. In contrast, ResNet-K requires a higher value, ie. ${C}_{max}=256$ to achieve maximum accuracy and recall. This indicates that the presence of MSA improves the learning ability of the model with a smaller ${C}_{max}$, allowing for constructing models with fewer parameters while improving prediction performance. However, as Figs. 7a, b indicate, MSAs degrade performance for larger models. These findings suggest that MSA is a valuable tool in model development, particularly for models with smaller ${C}_{max}$ values.

**Table 2:** Performance metrics of AlterNet-K and ResNet-K for various values of ${C}_{max}$ on the Rotterdam EyePACS AIROGS dataset

${C}_{max}$	AlterNet-K			ResNet-K		
	Accuracy	Recall	Parameter count	Accuracy	Recall	Parameter count
64	0.9084 ± 0.0065	0.8985 ± 0.0183	392,142	0.9032 ± 0.0053	0.8872 ± 0.0103	354,657
128	**0.9161** ± **0.0058**	**0.9070** ± **0.0069**	1,299,342	0.9000 ± 0.0051	0.8761 ± 0.0139	1,175,201
256	0.9160 ± 0.0104	0.9055 ± 0.0190	3,952,590	**0.9061** ± 0.0057	**0.8960** ± 0.0089	3,569,441
512	0.8898 ± 0.0149	0.8645 ± 0.0205	10,674,638	0.9042 ± 0.0090	0.8822 ± 0.0112	9,602,081

Bold values indicate the model that is giving the best results based on the Cmax parameter

*Effect of the number of intermediate stages in the AlterNet-K model *In this section, we describe the effect of the number of stages on the AlterNet-K model. Table 3 shows the performance of AlterNet-K128 and ResNet-K, each with 4 and 5 intermediate stages. AlterNet-K128 with 5 stages gives the best performance. AlterNet-K and ResNet-K models with 5 intermediate stages perform better than those with 4 stages. However, the 4-stage AlterNet-K128 outperforms both the 4-stage and 5-stage ResNet-K128, further reinforcing the findings in the previous section that MSA improves the learning ability of smaller models.

**Table 3:** Comparison of 5-stage models with 4-stage models on the Rotterdam EyePACS AIROGS dataset

Model	Number of stages	Accuracy	Recall	Parameter count
AlterNet-K128	4	0.9146 ± 0.0074	0.9015 ± 0.0056	974,757
AlterNet-K128	5	**0.9161** ± **0.0058**	**0.9070** ± **0.0069**	1,299,342
ResNet-K128	4	0.8940 ± 0.0076	0.8602 ± 0.0075	**879,777**
ResNet-K128	5	0.9000 ± 0.0051	0.8761 ± 0.0139	1,175,201

Bold values indicate the model that is giving the best result among 4 or 5 stage models

### Model interpretation

**Fig. 8:**
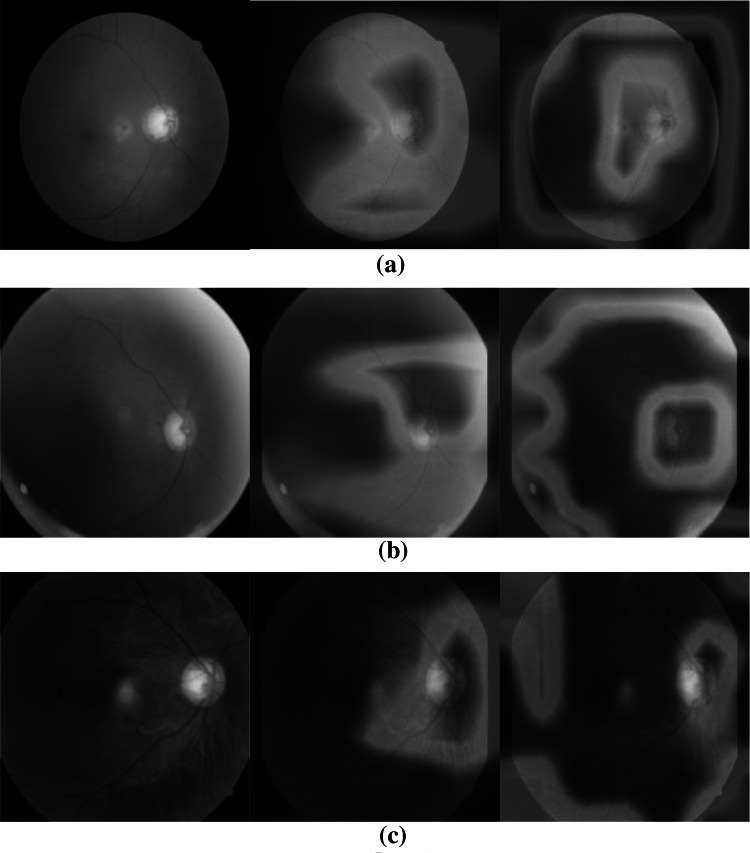
**a**–**c** CAMs generated by the AlterNet-K128 and ResNet-K128 models for sample images containing Glaucoma. Each sample shows an original fundus image (left), the corresponding CAM generated by ResNet-K128 (middle) and lastly, CAM generated by AlterNet-K128 (right)

CAMs are a powerful tool for visually interpreting the inner workings of CNN models. They help us understand whether the network is looking at appropriate parts of an image when making a prediction. Figure 8a–c shows CAMs for ResNet-K128 and AlterNet-K128 to highlight the effect of MSA. We only consider CAMs where the model makes a positive classification prediction for the given image. As seen in Fig. 8a, unlike ResNet-K128, which only activates near the OD region, AlterNet-K128 produces high activations in the OD and background regions of the image outside the eye. However, AlterNet-K achieves a better classification performance despite this anomalous activation in the background. Moreover, the CAMs produced by the pure CNN model ResNet-K are more diffuse around the OD. In contrast, the AlterNet-K model produces sharper activations near the OD.

## Conclusion

This work utilized deep learning methods to automatically identify Glaucoma in colour fundus images. A parameter-efficient AlterNet-K model was proposed. The model combines ResNets and MSAs in a way that leverages their complementary properties to improve the generalizability of the overall model. The AlterNet-K model outperformed novel transformer architecture such as ViT, Deit-S and Swin, standard DCNN models including ResNet, EfficientNet, MobileNet and VGG. However, the presence of MSA introduced localization anomalies in the CAMs generated by the model.

The results indicate that large CNN models are unnecessary for a high classification performance. Smaller models can achieve high classification accuracies when they are appropriately trained. Small and compact ResNet models combined with MSA outperform their larger counterparts. However, smaller, fully convolutional models, such as MobileNet, are not flexible enough to learn the necessary features for glaucoma identification. The models in this work can be expanded to handle classification tasks in other medical imaging domains, including several other eye diseases, such as cataracts and retinal diseases. It can also be applied to skin lesion classification. Since the AlterNet-K models are compact, they are easier to deploy to edge devices. The AlterNet-K models can be applied to larger datasets to study their properties further.
